# Semi-Quantitative vs. Volumetric Determination of Endolymphatic Space in Menière’s Disease Using Endolymphatic Hydrops 3T-HR-MRI after Intravenous Gadolinium Injection

**DOI:** 10.1371/journal.pone.0120357

**Published:** 2015-03-13

**Authors:** Georg Homann, Volker Vieth, Daniel Weiss, Konstantin Nikolaou, Walter Heindel, Mike Notohamiprodjo, Yvonne Böckenfeld

**Affiliations:** 1 Department of Diagnostic and Interventional Radiology, Eberhard-Karls-University, Tübingen, Germany; 2 Department of Clinical Radiology, University Hospital Münster, Münster, Germany; 3 Department of Otorhinolaryngology, Head and Neck Surgery, University Hospital Münster, Münster, Germany; Ohio State University, UNITED STATES

## Abstract

Magnetic resonance imaging enhances the clinical diagnosis of Menière's disease. This is accomplished by in vivo detection of endolymphatic hydrops, which are graded using different semi-quantitative grading systems. We evaluated an established, semi-quantitative endolymphatic hydrops score and with a quantitative method for volumetric assessment of the endolymphatic size. 11 patients with Menière's disease and 2 healthy subjects underwent high resolution endolymphatic hydrops 3 Tesla MRI with highly T2 weighted FLAIR and T2DRIVE sequences. The degree of endolymphatic hydrops was rated semi-quantitatively and compared to the results of 3D-volumetry. Moreover, the grade of endolymphatic hydrops was correlated with pure tone audiometry. Semi-quantitative grading and volumetric evaluation of the endolymphatic hydrops are in accordance (r = 0.92) and the grade of endolymphatic hydrops correlates with pure tone audiometry. Patients with a sickness duration of ≥ 30 months showed a significant higher total labyrinth fluid volume (p = 0.03). Fast, semi-quantitative evaluation of endolymphatic hydrops is highly reliable compared to quantitative/volumetric assessment. Endolymphatic space is significantly higher in patients with longer sickness duration.

## Introduction

Menière’s disease is a chronic disease with recurrent vertigo attacks, progressive hearing loss, tinnitus and/or aural fullness. Its etiology is still not fully understood, but the endolymphatic hydrops (EH) is considered as the morphological correlate for the symptoms. Several different, neither fully proven nor disproven theories exist regarding the formation of the endolymphatic hydrops: overproduction or an outflow obstruction of the endolymphatic fluid may influence the complex electrophysiological environment of the inner ear [1] and thereby the signal transduction of the cochlear and vestibular hair cells [2]. Some other theories regard ischemia as a cause, possibly stemming from vascular disorders [3].

Currently, Menière’s disease is not curable and the course of the disease, which can be unsteady and last for months up to decades, is not predictable. The unexpected occurrence, pronounced physical symptoms and the associated uncertainty accompanied by social restriction are perceived as extremely stressful by the patients [4]. Kirby et al. have shown, that the unpredictability and uncertainty of the disease process lead to a pronounced comorbidity concerning anxiety: Many patients develop general avoidance behaviour in relation to subjectively unsafe situations [5].

The recently established Endolymphatic Hydrops MRI (EH-MRI) allows the in-vivo diagnosis of EH and thereby assist in diagnosing Menières disease by use of i.v.-application of gadolinium-based contrast agents [6]. With this method the different inner ear compartments can be distinguished by variation of the inversion time in heavily T2 weighted FLAIR sequences resulting in positive perilymph images (PPI) and positive endolymph images (PEI). By post-processing using an image subtraction method proposed by Naganawa et al. [7] both anatomical information and the inner ear compartments can be displayed in one image series. Comparing the heavily T2 weighted FLAIR sequences to each other and to the total labyrinth volume assessed by T2 weighted cisternography, a grading of the extent of endolymphatic hydrops is possible.

Several different MR scoring systems for endolymphatic hydrops already exist. Currently, the magnetic resonance grading for endolymphatic hydrops itself has no significance in the diagnosis of Menière’s disease, but may be essential for the choice of therapy, follow-up and the evaluation of therapeutic success in the future, especially since recent studies demonstrated an enlargement of endolymphatic hydrops with longer sickness duration [8]. On the one hand the semi-quantitative analysis of the percentage of endolymphatic space in one slice allows a fast diagnosis [7,9]. On the other hand, scoring systems evaluating the perilymphatic space instead of the endolymphatic space [10] and quantitative single-slice analysis of endolymphatic and perilymphatic space have proven to be effective as well. Nevertheless, as could be demonstrated recently [11], special conditions of the inner ear (e.g. intralabyrinthine tumors with secondary Menière symptoms) may affect the single-slice-evaluation and the above mentioned methods for grading may therefore lose their diagnostic accuracy.

The objective of this study was to compare the results of a total quantitative measurement method of the endolymphatic space in Menière’s disease patients, in this case slice-by-slice volumetry with the results of those from semi-quantitative image evaluation and pure tone audiometry as functional parameter. Moreover, the question arises whether EH-MRI is able to assess subtle changes not only of endolymphatic but of the total lymph fluid due to sickness duration. Therefore, secondary target of this study was to assess these changes in our collective.

## Materials and Methods

### Ethics statement

The authors' institutional review board (Ethics committee of Münster University) has approved the conduct of this study. Written informed consent was given before every examination. Specific written consent for publication of the patients’ medical images included in the Figures was given.

### Patient Population

In this study 10 patients with definite and 1 patient with possible diagnosis of Menière’s disease according to the guidelines of the American Academy of Otolaryngology—Head and Neck Surgery [12] were included. Our department of otorhinolaryngology, head and neck surgery performed the clinical diagnosis, 2 healthy volunteers served as a control group.

The examination was carried out during a vertigo-free interval.

The patients age was 32 to 72 years (mean age: 54.91 years), 7 patients were female and 4 male.

### MR Imaging

MRI 4h after i.v.-injection of Gadolinium based contrast agents (GBCA) as has already been mentioned as a non-invasive tool for in vivo imaging of endolymphatic hydrops [13].

MR measurements were performed 4 hours after intravenous application of a single dose of Gadovist/Gd-DO3A-butrol (0.2 ml/kg or 0.1 mmol/kg body weight). The imaging was performed on a 3-tesla MR imaging unit (Philips Achieva, Philips Medical Systems, Best, NL) using a 16-channel array head and neck coil.

The evaluation of endolymphatic hydrops started with a T2DRIVE 3D MR Cisternography for anatomical reference of total lymph fluid. Acquisition parameters were: Repetition time (TR) of 2000 ms, echo time (TE) of 200 ms, flip angle of 90° and a refocusing angle of 120°. 1.0-thick axial slices covering the labyrinth were gained, FOV 100 x 100 x 37.5 mm, matrix size 240 x 161, voxel size 0.41 x 0.59 x 0.5 mm, acquisition time 4.5 minutes.

Afterwards, special sequences for differentiation of endolymphatic and perilymphatic fluid were adapted as proposed by Naganawa et al. [13]. Parameters for positive endolymphatic image (PEI; VISTA-IR-2050) were: Volumetric Isotropic T2w Acquisition (VISTA) sequence; TR 9000 ms; TE 540 ms; inversion time 2050 ms; SPAIR (Spectral Adiabatic Inversion Recovery) fat suppression pulse with an inversion delay of 220 ms; refocusing angle of 160°; matrix size 126 x 163; FOV 150 x 179.2 x 57.6 mm; slice thickness 1.0 mm; voxel size 1.1 x1.1 x 0.8 mm; acquisition time 9.5 minutes.

Parameters for positive perilymphatic image (PPI; VISTA-IR 2350) were: VISTA sequence; TR 9000 ms; TE 540 ms; inversion time 2350 ms; SPAIR fat suppression pulse with an inversion delay of 220 ms; refocusing angle of 160°; matrix size 126 x 163; FOV 150 x 179.2 x 57.6 mm; slice thickness 1.0 mm; voxel size 1.1 x 1.1 x 0.8 mm; acquisition time 9.5 minutes.

Total acquisition time (including anatomical T1-sequences) was 30–35 minutes.

After acquisition of the images, we obtained HYDROPS (HYbriD of Reversed image Of Positive endolymph signal and native image of positive perilymph Signal) images after motion correction by subtracting PEI from PPI as proposed by Naganawa et al. [14] on the side console (Achieva Philips Medical Systems, Best, Netherlands, Version 2.6). For subtraction results, negative signal values were allowed. On the HYDROPS image the perilymphatic space is suppressed, while the endolymphatic fluid is displayed as negative signal. The surrounding bone shows no signal.

### Image Assessment

The quality of the inner ear contrast enhancement was rated by two radiologists (3 and 15 years of neuroradiologic experience) in consensus using an ordinal 3-point-scale ranging from 0 to 2. 0 stood for no or insufficient inner ear contrast enhancement, 1 for reduced but diagnostically sufficient contrast enhancement and 2 for good contrast enhancement.

The gained sequences were semi-quantitatively analyzed on an EIZO workstation (EIZO Europe GmbH, Karlsruhe, Germany) using a GE PACS. The extent of endolymphatic hydrops was graded by two different radiologists (3 and 15 years of neuroradiologic experience), in consensus, according to the criteria of Nakashima, Naganawa et al. [7] on one single slice which displayed the vestibule or cochlea in maximum extent. Regarding the cochlea, a mild hydrops was defined by a slight displacement of Reissner’s membrane without exceeding the area of the scala vestibuli. The diagnosis of a significant endolymphatic hydrops required a bigger volume of the endolymphatic space than the residual scala vestibule. Where no endolymphatic hydrops was described, the Reissner membrane was not displaced at all. Concerning the vestibule, an endolymph-perilymph-ratio of more than one third was considered a mild hydrops and > 50% was considered a significant hydrops.

Volumetric evaluation was performed by one radiologist (3 years of neuroradiologic experience; blinded to the patient’s data) using slice-by-slice-segmentation of the outer margins of the labyrinth in MR-cisternography, and endolymphatic space in PEI using Amira 5.4 software (Visualization Sciences Group, Burlington, USA). The proportion of the endolymphatic space relative to the total labyrinth fluid space was expressed in percent and compared to the semi-quantitative analysis.

Evaluation time for both methods was measured, starting at the beginning of analysis and ending with the calculation of the endolymphatic space percentage (volumetric approach) or definition of the grade of endolymphatic hydrops (semi-quantitative analysis).

### Pure Tone Audiometry

On the day of examination, patients underwent standard pure tone audiometry (PTA) of each ear with values from 0.25 kHz to 8 kHz. As proposed by Seo et al [15] the gained values were averaged in the subdivisions LowTone_PTA_025_05 (0.25–0.5 kHz), MidTone_PTA_1_2 (1–2 kHz) and HighTone_PTA_4_8 (4–8 kHz) and afterwards correlated with the grade of endolymphatic hydrops, gained by volumetric assessment.

### Statistical Analysis

Statistical analysis was performed using SPSS 22 statistical software (SPSS Inc., Chicago, USA). Statistical dependence between grade of the endolymphatic hydrops and the pure tone audiometry was calculated by using Spearman’s rank correlation coefficient. The statistical significance of non-parametric data was tested with the Wilcoxon-Test and the parametric data with the T-Test. Values are given mean ± standard deviation, if not indicated otherwise.

For evaluation of the intra-method-reliability intra-observer testing was performed blinded, in randomized order and at different time points and expressed statistically as intra-class correlation (ICC) using the Spearman correlation coefficient.

## Results

### Inner Ear Contrast

No misregistration artifact from motion was noted in any image dataset of the ears. No patient was excluded from the analysis. No subject showed no or diagnostically insufficient inner ear contrast. The time between GBCA-injection and MR-measurement (on average 4.2 ± 0.4 h after injection) did not have an influence on the quality of the enhancement.

In two cases the inner ear contrast enhancement was graded with a score of 1 (reduced, but diagnostically sufficient contrast enhancement), while all other cases showed a good contrast enhancement (score of 2).

### Semi-quantitative vs. volumetric analysis

Semi-quantitatively for the total of n = 22 cochlear structures; in 12 inner ears no cochlear hydrops was detected, 4 inner ears presented a mild cochlear hydrops (I°) and 6 inner ears were affected significantly (II°) by cochlear endolymphatic hydrops. The quantitative analysis of the endolymphatic space yielded 3 inner ears with mild cochlear hydrops (I°), 6 with significant cochlear hydrops and 13 inner ears displayed no relevantly dilated endolymphatic space. Spearman’s rank correlation between both methods was ρ = 1 for the right ear and ρ = 0.95 for the left ear. The total correlation including vestibular and cochlear structures was ρ = 0.92.

**Fig 1:**
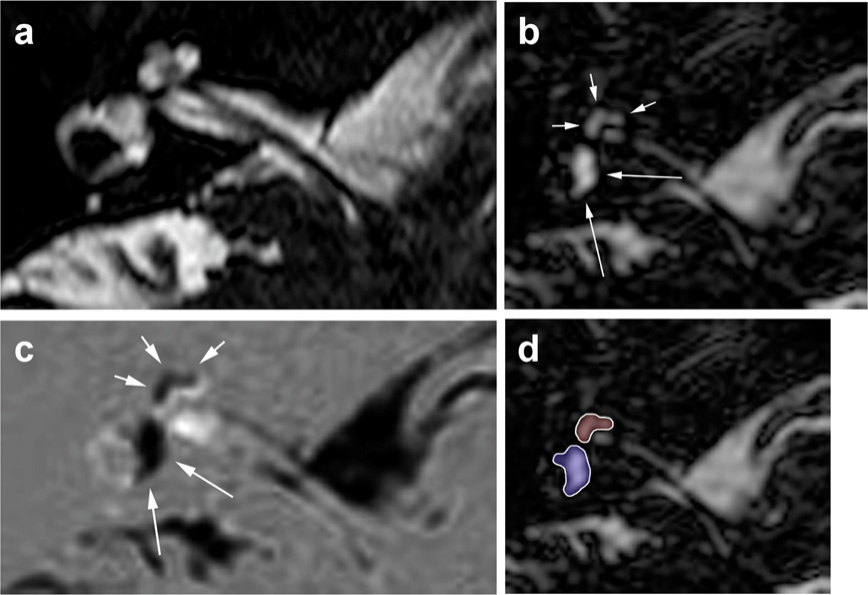
Images of a 57 year old woman with Menière’s disease, obtained 4h after i.v.-administration of a single-dose Gd-DO3A-butrol. (a) T2DRIVE-cisternography reveals the anatomy of the total lymphatic fluid of the right inner ear. (b) hT2w-FLAIR PEI with an inversion time of 2050 ms displays the endolymphatic fluid. (c) The HYDROPS (HYbriD of Reversed image Of Positive endolymph signal and native image of positive perilymph Signal) image, gained by subtraction of PEI from PPI, reveals the enlarged endolymphatic space as black area. Diagnosis after semi-quantitative analysis was a significant (II°) endolymphatic hydrops of the vestibule (long arrows) and the cochlea (short arrows). Volumetric assessment was performed by dedicated slice-by-slice-segmentation of the endolymphatic space in PEI (d) and the total labyrinth fluid space in T2DRIVE-cisternography. This patient showed an endolymph/total labyrinthine fluid-ratio of 50.51% (cochlea) and 54.3% (vestibule).

Fig. 1 shows an image example of a combined endolymphatic and perilymphatic hydrops.

**Fig 2:**
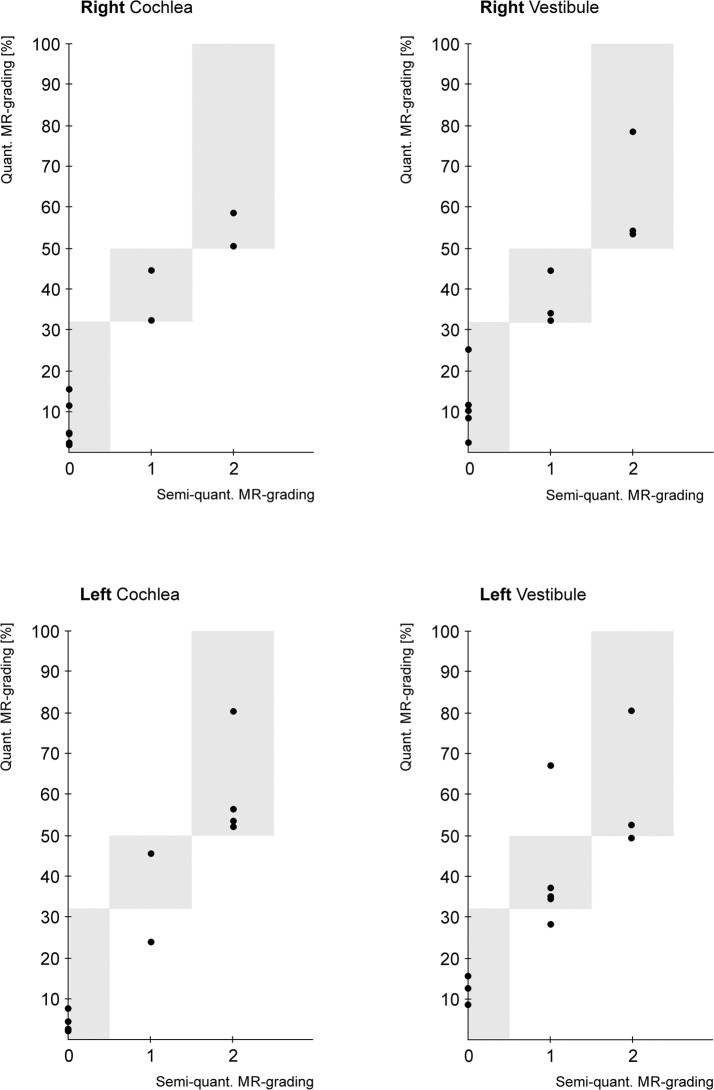
Comparison of volumetric [%] and semi-quantitative [0: No endolymphatic hydrops; 1: Mild endolymphatic hydrops; 2: Significant endolymphatic hydrops] evaluation of the degree of endolymphatic hydrops. The grey boxes reflect the grade margins of the volumetric approach (grade 0: < 33%; grade 1: 33–50%; grade 2: > 50%). The total correlation coefficient for vestibular and cochlear structures is 0.92. No significant difference was detected between volumetric and semi-quantitative approach (p > 0.05).

The semi-quantitative analysis of the vestibule presented the following results: 7 inner ears showed a mild vestibular endolymphatic hydrops (I°), 7 a significant vestibular EH (II°) and 8 inner ears showed no relevant EH (0°). Correlation between semi-quantitative and volumetric analysis were ρ = 1 for the right and ρ = 0.73 for the left vestibular structures (Fig. 2).

We detected no statistically significant difference for either method. Intra-observer testing for volumetric analysis resulted in an ICC of 0.99.

No significantly dilated endolymphatic space could be documented for the asymptomatic subjects.

Average analysis time for the volumetric assessment was 14.5 minutes per ear (± 2.81), whilst the semi-quantitative approach took 2.2 minutes (± 0.8). The difference was statistically significant (p < 0.05; T-Test).

### Labyrinth Volume

**Fig 3:**
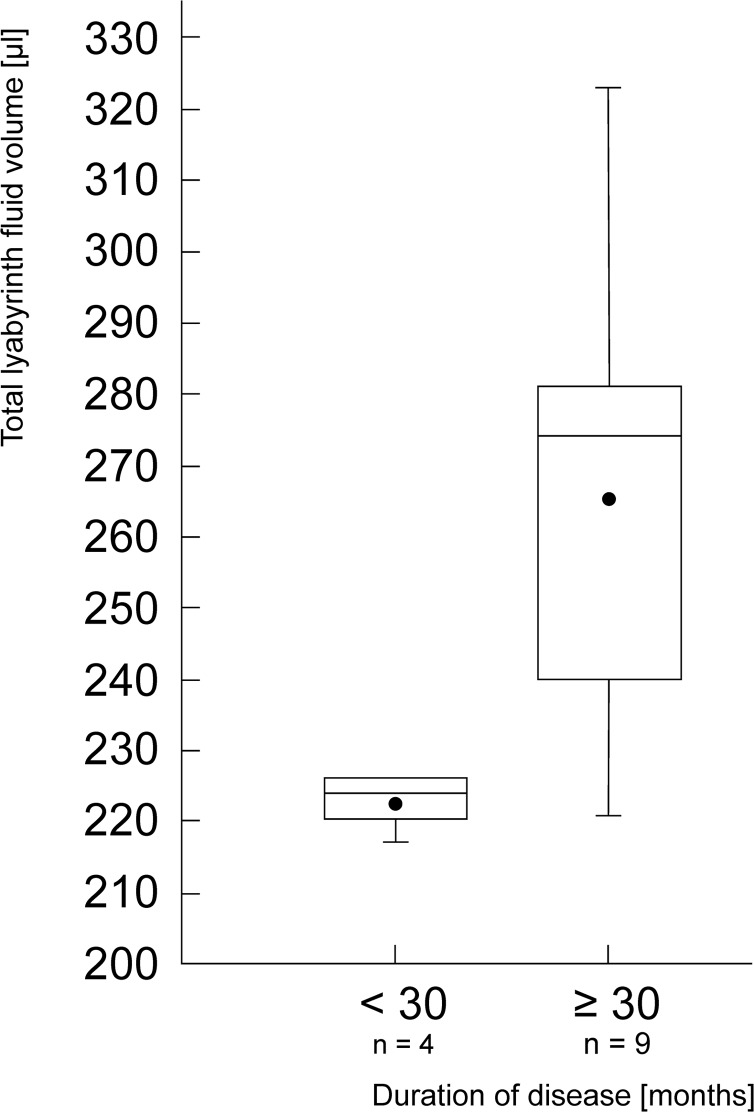
Influence of the duration of disease on the total labyrinth fluid volume in ears affected by endolymphatic hydrops. The box depicts the lower and upper quartile as well as the median; the black dot represents the mean. The whiskers depict a maximum and minimum of 1,5x of the interquartile range, statistical outliers are expressed as small circles. The difference between both groups was considered significant (p < 0.05).

The volumetric measurements of the inner ear liquid compartments showed a mean volume of the cochlear labyrinth of 102 μl (range: 82–118 μl) and a mean volume of the vestibular labyrinth (including the semicircular canals) of 162.73 μl (range: 127–220 μl). The total labyrinth fluid mean volume was 264.73 μl (range: 216–326 μl). Regarding the sickness duration, the mean affected labyrinth volume of patients with sickness duration < 30 months (n = 4) was 222.75 μl and 265.44 μl for those with ≥ 30 months (n = 9). The difference was statistically significant (p = 0.03; Wilcoxon-Test; Fig. 3).

### Pure Tone Audiometry and clinical correlation

Symptoms occurred in the left ear in 5 cases, in the right ear in 4 cases and in one case both ears were affected.

**Fig 4:**
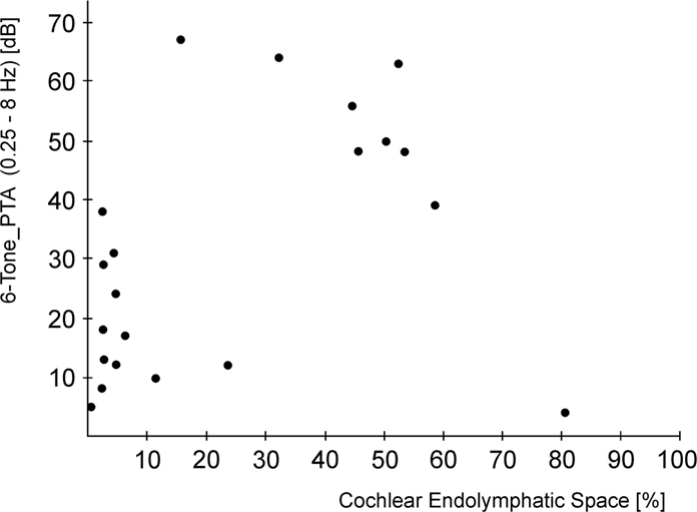
Correlation between 6-tone pure-tone-audiometry (6-Tone_PTA) and percentage of vestibular endolymphatic space to total labyrinth fluid (Vestibular endolymphatic space/total labyrinth volume x 100).

Affected ears were defined as ears with MR-diagnosis of a cochlear endolymphatic hydrops. The PTAs of the 11 patients demonstrated an overall average hearing level (6-Tone_PTA) of 44 ± 18.64 dB in the affected ears and 21 ± 17.1 dB in the unaffected ears. The difference threshold of the affected and the non-affected ear was significant (p = 0.011; T-Test; Fig. 4). The correlation between the grade of cochlear endolymphatic hydrops and the frequency-specific subdivided PTA-levels was found to be good for the low (ρ = 0.572) and mid-tone-spectrum (ρ = 0.542), whilst the high tone PTA did not correlate well with the MR-grade (ρ = 0.233). The correlation for LowTone_PTA and MidTone_PTA was statistically significant (p < 0.05; T-Test).

## Discussion

We were able to show that established semi-quantitative MR evaluation and volumetry of endolymphatic hydrops by magnetic resonance imaging after i.v.-administration of Gadolinium-based contrast agents (GBCA) both are relevant and useful for the diagnosis of endolymphatic hydrops. Moreover, we were able to show that the semi-quantitative grading system proposed by Naganawa et al. [7], which can be deployed relatively quickly, is reliable.

As the proposed volumetry of the inner ear fluid compartments is a very time-consuming procedure, its clinical application has only been performed scientifically and in selected cases as with the occurrence of inner ear tumors, so far. Since the first report of MR-volumetry of the inner ear liquids [11], a semi-automated volumetry algorithm has been invented [16], which may reduce the total evaluation time. In our daily practice however, only the semi-quantitative approach was applied and only in unclear cases, this procedure was occasionally enhanced by the proposed volumetry. There are, nevertheless, several good reasons to believe that follow-up evaluation with relatively small changes of the endolymphatic volume may be assessed more precisely via total inner ear volumetry: Especially with regard to recent publications on pharmaceutical effects on the size of the endolymphatic hydrops with small or nearly non-detectable changes [17], we expect a higher accuracy and comparability by a dedicated volumetric approach. Hypothetically, small changes in endolymph fluid volume may provide evidence for therapy response or non-response and progression of endolymphatic hydrops under therapy could thereby be detected earlier.

In contrast to intratympanic application of GBCA, the intravenous method allows a much faster and more reliable approach. Especially considering recent studies, which have demonstrated that the contrast agent distribution via the round window is altered in Menière’s disease [18] and the success rate of intralabyrinthine gadolinium distribution is thereby reduced. Patients affected by Menière’s disease may thereby profit from i.v.-application. The i.t.-administration, due to its non-systemic distribution, is practical in patients with impaired renal function, so both methods should be taken into consideration for individual application.

Before the introduction of Endolymphatic Hydrops MRI the imaging findings focused on morphological abnormalities ranging from abnormal periaqueductal pneumatisation [19] to hypoplasia of the retrovestibular bone structures [20], which lead to the hypothesis of developmental abnormalities as enabling factors for the emergence of Menière’s disease. Since Menière’s disease appears to have a multifactorial genesis and its course is influenced by several parameters, we refocused on the volumetric parameters of our study population:

Compared to other MRI and histological studies on healthy subjects [16,21,22] the labyrinthine volumes of our affected population seemed slightly elevated. This may have been because the dedicated slice-by-slice segmentation allows the full record of the labyrinthine volume, especially of the semicircular canals, in comparison to other MRI studies using semi-automated procedures. Moreover, in contrast to computed tomography studies focused on the bony labyrinth [23] we detected a significant increase of the total labyrinth fluid volume relative to the duration of the disease which may hypothetically be based on pressure-dependent dilatation. This hypothesis is underpinned by a recently reported case with increasing endolymphatic space over the course of two years [8]. To evaluate possible implications of the enlarged labyrinth volume on the course of the disease, further MR-investigations are needed.

A recent publication by Liu et al., conducted with healthy volunteers [24] using a semi-quantitative, single-slice approach found a normal endolymphatic portion for the cochlea of 8–26% and 20–41% for the vestibule and thereby defined the upper margins of this normal collective as a threshold for diagnosis of endolymphatic hydrops. These numbers differ from the chosen thresholds in this study, which were adapted from the currently most-applied semi-quantitative grading-system by Nakashima. Due to ethical guidelines we were not able to include more healthy subjects to be able to define specific thresholds for the volumetric approach. However, regarding the high grade of accordance between our semi-quantitative analysis and the volumetric approach, it can be assumed that the single-slice-approach by Liu et al., with a central position of the measurement slice and therefore exclusion of the peripherally localized structures (especially the semicircular canals), leads to an overestimation of the endolymphatic volume in this specific slice. We therefore propose further population studies to define the normal endolymphatic volume and its variance. This is gaining more and more importance, since it is a common finding that not only the clinical affected side shows endolymphatic hydrops, but the contralateral ear as well [25] and causality is still unclear.

Our study is essentially limited because of the low number of included patients. Nevertheless, we were able to compare both methods and we gained significant results. Furthermore, we did not gain histological confirmation of endolymphatic hydrops, because the acquisition of histological specimens, in benign conditions as Menière’s disease is currently not possible. Moreover, the size of the control group (n = 2) was very small. Since recent studies have shown that endolymphatic hydrops may also occur in healthy subjects [24], the demand for bigger demographic studies using magnetic resonance tomography is underpinned.

Correlating with the findings of Naganawa et al. [9], the evaluation of the endolymphatic volume on PEI may lead to a slight underestimation of the percentage of endolymphatic space.

When considering that the existing clinical methods to prove Menière’s disease lack efficiency and sensitivity, Endolymphatic Hydrops MRI becomes a highly recommendable diagnostic tool. It may enhance the well-known methods of electrocochleography and glycerol testing which have an estimated sensitivity of 60–70% [26,27]. That means that the prevalence of positive testing is relatively small and a negative result is almost negligible. Thus, in clinical routine these tests did not gain broad acceptance and the main criterion for the diagnosis of Menière’s disease is the clinical appearance following the guidelines of the American Association of Otorhinolaryngology, Head and Neck Surgery [12]. The pitfall in a purely clinical diagnosis is that a certain group of patients with Menière’s disease or Menière-like symptoms could be diagnosed false positive and hence the therapy may be insufficient.

In summary, our study shows that the existing semi-quantitative grading systems are highly accurate, and only special anatomical conditions (e.g. intralabyrinthine tumors, anatomical variants [11]) may profit from additional evaluation by volumetry. Compliant with other studies we could not only detect endolymphatic hydrops, but could prove that cochlear endolymphatic hydrops correlates with loss of cochlear function [15,17], which underpins the usefulness of endolymphatic hydrops MRI in the evaluation of Menière’s disease.

## Supporting Information

S1 Dataset Patient characteristics and MRI analysis results. The table displays the evaluation data of the semi-quantitative and volumetric assessment of endolymphatic hydrops.
(XLSX)

S2 Dataset Pure-tone audiometry results. The table shows the results of the pure-tone audiometry (raw data).
(XLSX)
